# Salmagundi

**Published:** 1888-04

**Authors:** 


### SALMAGUNDI.

EDITED BY E. G. BETTY, D.D.S.

### Echoes from the M. V. D. A.

Dr. C. Whaley, of Pomeroy, was present and had the good sense to bring his estimable wife.

The meeting just held was one of the breeziest and most interesting during the last ten years or more, every one seeming to take a personal interest in all that went on.

If the attendance keeps on increasing at the rate it has done for several years past, the college authorities will have to let out a seam in the auditorium to accommodate the membership.

Dr. C. M. Wright, Chairman of the Executive Committee, is certainly the write man in the rite place for he is a right good hustler from away back; *q. v.* the amount of writing material present.

A PRIVATE letter to the Editor of SALMAGUNDI from the mother of Dr. A. G. Rose, our Secretary, states that he has rapidly improved, a fact that many inquiring friends will be pleased to know.

---

IT appears that the near approach of the annual meeting of the American Medical Association kept very few of the "stand-bys" away; so much to the credit of the Executive Committee and its excellent programme.

---

IT is a pleasure to know that the Society reconsidered its action upon the resolution, inspired by the letter of its President, Dr. Harlan, regarding the custom of some of our colleges in rushing through foreigners for practice abroad, thus avoiding the stigma of stultification.

---

WHILE we greatly favor the introduction of new methods and appliances, we nevertheless must protest against the M. V. D. A. granting so much of its time and attention in the way of a "special order of business" to any one who has an ax to grind, and heartily agree with Dr. Conrad, of St. Louis, that the "clinic's the thing."

---

CONTRARY to expectation and greatly to the surprise and gratification of all, Dr. Atkinson made his appearance and went into the debates with his own peculiar vim and energy, to the edification of some and the enjoyment of everybody. There is something about Atkinson which stamps him as a peculiar genius, (and we say this reverently) far above the average intellectually, and which attracts his hearers in spite of themselves. Long may he live and may his shadow never grow less.

---

ONE thing we would like to impress upon the minds of the graduates of the Ohio College as they go forth each year, is the fact that they owe it to themselves to enrol their names on the roster of the M. V. D. A., the oldest Dental Association in the world, and in years after they can look back with pride to the fact that they had the honor of becoming members thereof, at the outset of their careers.

As the years pass by we notice that the old stagers reproduce themselves in their sons. Some attend the meetings to introduce the boys, others send them as representatives, others again have passed away, leaving their lineal descendants to carry on the revered name. Thus we see the procession move along and wonder when our time will come, Our good old friend Dr. J. W. Baxter, of Vevay, Ind., lives again in the person of his son, J. W. Jr.; Prof. Taft may be proud of his Will.; Dr. G. W. Keely of Charlie, our friend and classmate; Prof. H. A. Smith has just graduated his son and probable successor; Atkinson, and there is but one, left his boy at home "to keep shop"; McKellops—our own dear Mac—has turned his boy into a bibliographer, and while he himself goes abroad to attend the meetings and gather up back numbers when he is not engaged in getting contributions to fight some monopoly in the meanwhile; brother Conrad bets that it *will* be a boy, in which hope we will rest pending developments.

"Nothing is more noteworthy than the remarkable testimony of the almost universal possession of good digestion by those who live to a good old age."

Medicine a Mean Trade.—The practice of medicine must be very disappointing to those who follow it chiefly for the acquisition of wealth. Whoever practices it in a commercial spirit debases the calling and degrades himself. As a French writer has truly said: "Medicine is the noblest of professions, but the meanest of trades."—*Dr. Cotting.*

"The researches on the breath of man and other animals, by Brown Sequard and d'Arsonval, make it evident that the air exhaled by them, even in a healthy state, contains a very powerful toxic element, apart from $C O_2$, to which probably is due the bad effects caused by breathing a close atmosphere; and further investigations lead to the conclusion that this volatile organic poison is an alkaloid. Its molecular composition has not yet been determined."

